# Family History of Education Predicts Eating Disorders across Multiple Generations among 2 Million Swedish Males and Females

**DOI:** 10.1371/journal.pone.0106475

**Published:** 2014-08-27

**Authors:** Anna Goodman, Amy Heshmati, Ilona Koupil

**Affiliations:** 1 CHESS, Centre for Health Equity Studies, Karolinska Institutet, Stockholm University, Stockholm, Sweden; 2 Faculty of Epidemiology and Population Health, London School of Hygiene and Tropical Medicine, London, United Kingdom; RWTH Aachen University, Germany

## Abstract

**Purpose:**

To investigate which facets of parent and grandparent socio-economic position (SEP) are associated with eating disorders (ED), and how this varies by ED subtype and over time.

**Methods:**

Total-population cohort study of 1,040,165 females and 1,098,188 males born 1973–1998 in Sweden, and followed for inpatient or outpatient ED diagnoses until 2010. Proportional hazards models estimated associations with parental education, income and social class, and with grandparental education and income.

**Results:**

15,747 females and 1051 males in our sample received an ED diagnosis, with rates increasing in both sexes over time. ED incidence in females was independently predicted by greater educational level among the father, mother and maternal grandparents, but parent social class and parental income showed little or no independent effect. The associations with education were equally strong for anorexia nervosa, bulimia nervosa and ED not-otherwise-specified, and had increased over time. Among males, an apparently similar pattern was seen with respect to anorexia nervosa, but non-anorexia ED showed no association with parental education and an inverse association with parental income.

**Conclusions:**

Family history of education predicts ED in gender- and disorder-specific ways, and in females the effect is observed across multiple generations. Particularly given that these effects may have grown stronger in more recent cohorts, these findings highlight the need for further research to clarify the underlying mechanisms and identify promising targets for prevention. Speculatively, one such mechanism may involve greater internal and external demands for academic success in highly educated families.

## Introduction

Eating disorders (ED) are an important cause of morbidity and mortality [1]–[3], with anorexia nervosa having the highest mortality rate of any mental disorder [4]. Several British and Swedish studies also suggest that hospitalisation for ED may be unusual among physical and mental disorders in showing a positive association with higher family socio-economic position (SEP) [5]–[7], although other population-based studies (based upon fewer ED cases) report mixed or null results [8]–[10]. There thus remains some uncertainty as to whether, or in what settings, high SEP predicts an increased risk of ED, but the potential importance of this issue is highlighted by the large population health impacts that might plausibly accompany any effect. For example, one recent large Swedish study reported a population attributable fraction of 24% between mother's education and daughter's ED [5]. Further understanding of the nature and causes of this association, and how any association is changing over time, may inform the design of public health prevention programmes.

Most previous large studies (>500 cases) have, however, focussed either upon anorexia nervosa alone [6], [7] or else upon ED as a group [5]. This has prompted the critique that such studies could distract from or obscure a possible negative association between SEP and non-anorexia ED (e.g. bulimia nervosa) [11], [12]. These critiques have further suggested that any positive associations with SEP have weakened over time [11], but previous studies have not covered a long enough time period to study this directly. Previous quantitative studies have also been limited in typically only including single SEP indicators, and typically only including females. Examining whether some facets of SEP show stronger associations than others, and how these differ across the sexes, could provide clues regarding the underlying mechanisms [13], [14]. Finally most previous studies have only measured family SEP in terms of parental characteristics. Nevertheless a recent ethnographic study argued that socio-cultural influences on ED may reflect the accumulated SEP experience of multiple generations [15]. Only one, small quantitative study has examined this issue (N = 53 ED cases), and reported suggestive evidence of an association between maternal grandmother's education and ED in Swedish females [16].

This paper therefore used a large, total-population Swedish cohort to examine whether associations between parental SEP and offspring ED are stronger (i) for some socio-economic indicators than others, (ii) for some disorder subtypes than others and (iii) for those born earlier versus later in our cohort. We then examined whether grandparental SEP showed any associations with ED risk, independent of parental SEP. Mindful of the potential for bias if socio-economically advantaged groups seek or access care for more mild disorders, we also tested the hypothesis that incident inpatient cases from high socio-economic groups would have a shorter duration of hospitalisation. In this we followed one previous British study [6], which has used this measure as an (admittedly imperfect) marker for disorder severity at the point of accessing healthcare.

## Methods

### Study population

We used Swedish register data to create a total-population cohort of individuals born 1973–1998, and followed them up for ED from 1985–2010. As migration data were not available from 2003–2010, we restricted our study population to Swedish-born offspring of Swedish-born parents, a group with very low emigration rates. This restriction also maximised data completeness and reduced the potential for residual confounding. After excluding those who were adopted (0.2%), died (0.8%) or emigrated (0.3%) our study population comprised 1,040,165 females and 1,098,188 males. Ethical approval was granted by the Regional Ethics Committee in Stockholm, Sweden, and all data were fully anonymised and de-identified prior to analysis.

### Eating disorder diagnoses

We used International Classification of Disease (ICD) codes [17] to define ED as ICD-8 code 306.5 (available 1985–1986), ICD-9 codes 307.1 and 307.5 (available 1987–1996) and ICD-10 codes F50.0–F50.9 (available 1997–2010). When analysing ED subtypes in females, we distinguished anorexia nervosa (F50.0–F50.1), bulimia nervosa (F50.2–F50.3), and eating disorder not otherwise specified (EDNOS, F50.4–F50.9). This three-way division was not possible prior to ICD-10. To avoid double-counting disorders, we excluded from our analyses of EDNOS any diagnoses made within 1 year of a diagnosis of anorexia or bulimia nervosa, assuming that these represented the same underlying disorder [1], [18]. When analysing ED subtypes in males we only distinguished between anorexia nervosa (307.1, F50.0–F50.1) and non-anorexia ED in order to allow us to increase power by using both ICD-9 and ICD-10 diagnoses.

We identified cases as individuals with a main or a secondary ED diagnosis in the Swedish national inpatient, outpatient or death registers. These provide national data of high quality, with validation studies indicating that in general the positive predictive values of psychiatric diagnoses range from 85–95% [19]. The inpatient and death registers were available throughout follow-up, with high national inpatient coverage in 1985–6 (≈95%) rising to near-complete coverage from 1987 onwards (>99%). This included coverage of psychiatric hospital discharges from both public and private facilities. By contrast outpatient register information was only available since 2001 and generally lacked information from private providers, giving an overall coverage of around 80% [19]. Our findings were very similar in sensitivity analyses restricted to inpatient register diagnoses.

### Parent and grandparent characteristics

The socio-economic indicators we examined are presented in Table 1. Parents' and grandparents' highest educational level was identified as the highest ever recorded in the census (1960–1990) or the Education register (1985–2001). Income was derived from the 1970 and 1990 censuses, by (i) standardising household income by age and sex and (ii) averaging these standardised incomes across couples (e.g. across maternal grandmothers and grandfathers). Parental social class was assigned as the highest social class between mothers and fathers, using head of household social class in the 1990 census if available (97%) or individual social class in the 1980 census if this was missing (3%).

**Table 1:** Parental and grandparental predictors of rates of eating disorder hospitalisation among Swedish females born 1973–1998 (15 747 cases in 1,040,165 individuals).

			No. females	No. ED cases	Rate per 100 000	Adjusted hazard ratio (95% CI)
Paternal	Grandfather's	Basic	616,664	8996	106.6	1
grand-	highest	Secondary	256,918	3960	145.1	1.01 (0.97, 1.06)
parents	education level	Tertiary	97,670	1697	167.3	1.02 (0.96, 1.08)
		Post-graduate	7042	152	214.0	1.14 (0.97, 1.35)
	Grandmother's	Basic	653,489	9670	104.9	1
	highest	Secondary	274,850	4255	149.9	1.04 (1.00, 1.08)
	education level	Tertiary & above	84,028	1406	191.8	1.04 (0.97, 1.11)
	Grandparents' income	Change per standard deviation	1,019,650	-	-	0.99 (0.97, 1.01)
Maternal	Grandfather's	Basic	601,326	8586	102.7	1***
grand-	highest	Secondary	286,591	4395	141.3	1.03 (0.99, 1.07)
parents	education level	Tertiary	103,561	1934	178.6	1.11 (1.05, 1.18)
		Post-graduate	6939	162	233.0	1.24 (1.05, 1.45)
	Grandmother's	Basic	619,007	9036	101.4	1**
	highest	Secondary	312,674	4772	144.1	1.03 (0.99, 1.07)
	education level	Tertiary & above	94,583	1743	206.0	1.12 (1.05, 1.19)
	Grandparents' income	Change per standard deviation	1,031,782	-	-	1.01 (0.99, 1.02)
Parents	Father's highest	Basic	230,971	2983	85.9	1***
	education level	Secondary	516,099	7253	115.9	1.08 (1.03, 1.13)
		Tertiary, <3 years	132,572	2283	146.6	1.22 (1.15, 1.30)
		Tertiary, ≥3 years	146,151	2887	155.3	1.28 (1.20, 1.36)
		Post-graduate	13,934	336	181.5	1.42 (1.25, 1.61)
	Mother's highest	Basic	147,919	1893	83.2	1***
	education level	Secondary	549,062	7597	111.3	1.03 (0.98, 1.08)
		Tertiary, <3 years	172,884	2933	142.8	1.12 (1.05, 1.19)
		Tertiary, ≥3 years	165,887	3203	150.6	1.17 (1.09, 1.25)
		Post-graduate	4157	118	226.4	1.51 (1.24, 1.83)
	Parents' income	Change per standard deviation	1,040,153	-	-	1.00 (0.98, 1.02)
	Parents'	Unskilled manual	136,871	1885	110.0	1**
	occupational	Skilled manual	193,503	2588	113.4	0.99 (0.93, 1.04)
	social class	Farmer/self-employed	148,608	1998	106.0	0.99 (0.93, 1.05)
		Low non-manual	79,153	1032	92.7	0.98 (0.91, 1.06)
		Medium non-manual	262,882	4262	125.3	1.07 (1.01, 1.14)
		High non-manual	195,181	3609	135.2	1.08 (1.01, 1.15)

*p<0.05, **p<0.01, ***p<0.001 for heterogeneity. CI = confidence interval. Basic education is up to age 16, Secondary education is up to age 18–19. Adjusted analyses adjust for all variables in the column plus the age of the grandparents and parents at the birth of their daughter, and the birth year of the index cohort member. See Table S1 in File S1 for extended versions of this table that include minimally-adjusted and intermediate regression models.

### Duration of hospitalisation

For those ever receiving inpatient diagnoses of ED, we calculated the duration (in days) of their first hospitalisation, combining periods spent in different hospital units as part of the same uninterrupted treatment period. We followed standard practice in assigning a value of ‘1 day’ to patients admitted and discharged on the same day [19].

### Statistical analysis

We used Cox proportional hazards regression models to calculate hazard ratios, starting follow-up when the child turned 12 and continuing until 31 December 2010 or until death, emigration or first diagnosis for the outcome in question (whichever was earliest). When examining predictors of ED subtypes, we did not censor individuals receiving a different ED diagnosis (e.g. when bulimia nervosa was the outcome we did not censor individuals diagnosed with anorexia nervosa).

In adjusted analyses, we included not only SEP variables, but also the child's birth year and the age of the parent or grandparent when the child was born. All regression models entered continuous variables as linear terms, including quadratic terms if these showed evidence (p<0.05) of non-linearity in adjusted analyses. When analysing associations with ED in males, we *a priori* banded education and social class categories into larger groups, in order to reduce random variation due to small cell sizes.

The percent missing data ranged from 0–6% across the parental and grandparental characteristics. We used multiple imputation (five imputations) to impute these data under an assumption of missing at random, including in our imputation model event indicators for the outcome and the Nelson-Aalen estimator of cumulative hazard [20]. Our substantive findings were unchanged in analyses restricted to individuals with complete data. All analyses used Stata 11.

## Results

### Incidence of eating disorders

By the end of 2010, 15,747 females (1.5%) and 1051 males (0.1%) had received an ED diagnosis, 4177 from the inpatient register (available 1985–2010) and 12,621 from the outpatient register (available 2001–2010). Univariable Cox models indicated that the rate of first ED diagnosis in the inpatient register increased somewhat over time among females, particularly in the earlier part of the time period (HR 1.22 (95% CI 1.14, 1.30) for those born 1982–1989 vs. 1975–1981; HR 1.07 (95% CI 0.99, 1.16) for those born 1990–1998 vs. 1982–1989). In males the rate was unchanged in those born 1982–1989 vs. 1975–1981 (HR 0.98, 95% CI 0.74, 1.29) but then increased in the cohort born 1990–1998 (HR 1.54 (95% CI 1.17, 2.04) for those born 1990–1998 vs. 1982–1989).

### Parental SEP and ED risk in daughters

**Figure 1:**
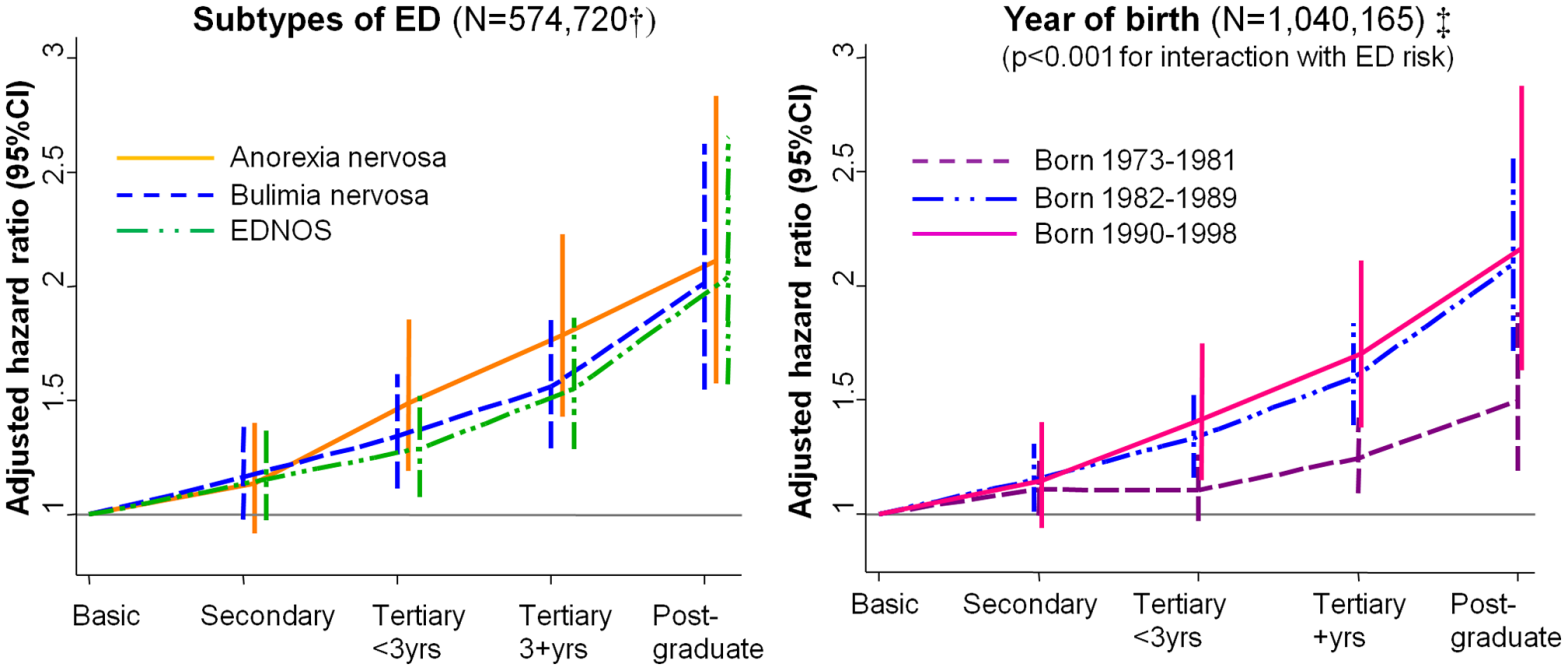
Associations between parental educational level and ED in daughters, comparing associations across ED subtypes and across birth years. CI = confidence interval, ED = eating disorder, EDNOS = eating disorder not otherwise specified. All models adjust for all parent SEP variables (Table 1) plus parental age and the index cohort member's birth year. †Analyses comparing ED subtypes exclude those born before 1985 because the three-way division of these subtypes only became possible in 1997 when ICD-10 was introduced. N = 4362 females for anorexia nervosa; N = 1169 for bulimia nervosa; and N = 4607 for EDNOS. ‡Results very similar if restricted to inpatient diagnoses, which were available across all years for all cohort members.

Mother's and father's education were strongly and positively associated with ED in their daughters, with a two to three-fold difference in the crude ED rate between parents educated to postgraduate level versus those who left school at age 16. After adjusting for other parent and grandparent characteristics, there remained strong evidence of independent, dose-response associations with both mother's and father's education (Table 1). This association with parental education was observed for anorexia nervosa, bulimia nervosa and EDNOS, and the magnitude of the association was similar across these three subtypes (Figure 1, left side; see also Table S2 in File S1). This association was also observed across females born in different years, but the effect was somewhat stronger in the younger cohorts examined (p<0.001 for interaction between birth year and education: Figure 1, right side).

After adjusting for parental education, there was no evidence that parental income independently predicted ED or any ED subtype among females (all p>0.05). The effect of parental occupational social class was also substantially attenuated upon adjusting for education, although in this case some evidence remained of a relatively modest increased risk associated with high or medium non-manual class (Table 1, Table S1 in File S1). Comparisons across the different ED subtypes suggested that this remaining independent effect of social class was driven by an association with anorexia nervosa (e.g. adjusted HR for high non-manual versus unskilled manual social class 1.32 (95% CI 1.15, 1.50) for anorexia nervosa versus 1.01 (0.78, 1.30) for bulimia nervosa and 1.00 (0.88, 1.13) for EDNOS; see Table S2 in File S1).

### Parental SEP and ED risk in sons

Whereas the different ED subtypes showed very similar patterns of association with SEP in females, in males there appeared to be a difference between anorexia nervosa and non-anorexia ED. Specifically, anorexia nervosa again showed a trend towards a positive association with parental education (albeit not reaching statistical significance) and showed little or no association with the other SEP indicators (Table 2). By contrast, non-anorexia ED was largely unrelated to parental education and inversely associated with income (all p<0.004 for sex interaction). Note that the large majority of these non-anorexia cases of ED seemed likely to be EDNOS and not bulimia nervosa; male cases of the former outweighed the latter by almost 15∶1 (399 vs. 26) among those born after 1985, for whom ICD-10 codes were always available. There was no evidence that these effects varied according to year of birth (all p>0.2 for interaction), but these tests for interaction were unfortunately substantially underpowered.

**Table 2:** Parental and grandparental predictors of rates of eating disorder hospitalisation among Swedish males born 1975–1998.

			No.	Anorexia nervosa			Non-anorexia nervosa ED		
			males	No. cases	Rate per 100 000	Adjusted hazard ratio (95% CI)	No. cases	Rate per 100 000	Adjusted hazard ratio (95% CI)
Paternal	Grandfather's	Basic	588,145	225	3.0	1	357	4.8	1
grand-	highest education	Above basic	365,293	164	4.5	1.04 (0.82, 1.32)	225	6.1	0.98 (0.82, 1.18)
parents	Grandmother's	Basic	619,073	233	2.9	1	361	4.5	1
	highest education	Above basic	365,418	171	4.9	1.20 (0.96, 1.51)	245	7.0	1.17 (0.97, 1.40)
	Grandparents' income	Change per standard deviation	990,690	-	-	0.90 (0.77, 1.04)	-	-	1.01 (0.98, 1.04)
Maternal	Grandfather's	Basic	569,881	212	2.9	1	332	4.5	1
grand-	highest education	Above basic	400,183	186	4.5	1.21 (0.96, 1.51)	265	6.5	1.11 (0.92, 1.32)
parents	Grandmother's	Basic	580,891	236	3.1	1	348	4.5	1
	highest education	Above basic	413,909	174	4.3	0.92 (0.74, 1.14)	268	6.6	1.05 (0.87, 1.25)
	Grandparents' income	Change per standard deviation	999,377	-	-	0.98 (0.88, 1.10)	-	-	0.99 (0.88, 1.11)
Parents	Father's highest	Basic	212,097	76	2.6	1	130	4.4	1
	education level	Secondary	504,973	190	3.3	1.00 (0.76, 1.32)	307	5.4	1.00 (0.81, 1.23)
		Tertiary or higher	287,744	146	4.4	1.22 (0.88, 1.69)	178	5.3	1.07 (0.82, 1.39)
	Mother's highest	Basic	133,351	39	2.1	1	89	4.8	1
	education level	Secondary	534,101	209	3.4	1.26 (0.89, 1.79)	316	5.1	0.87 (0.69, 1.11)
		Tertiary or higher	337,576	164	4.2	1.38 (0.94, 2.04)	211	5.4	0.94 (0.71, 1.25)
	Parents' income	Change per standard deviation	1,005,304	-	-	1.03 (0.96, 1.11)	-	-	0.82 (0.72, 0.95)***
	Parents' occupational	Manual/famer/self-employed	398,827	154	3.3	1	256	5.5	1
	social class	Non-manual	583,284	252	3.6	0.92 (0.72, 1.16)	342	4.8	0.98 (0.80, 1.19)

*p<0.05, **p<0.01, ***p<0.001 for heterogeneity. ED = eating disorder not-otherwise-specified, CI = confidence interval. Basic education is up to age 16, Secondary education is up to age 18–19. Adjusted analyses adjust for all variables in the column plus the age of the grandparents and parents at the birth of their son, and the birth year of the index cohort member. Note that the birth years are restricted to 1975–1998, as it is for males born in in these years that the distinction between anorexia and non-anorexia ED was always available in the contemporary ICD version.

### Grandparental SEP

**Figure 2:**
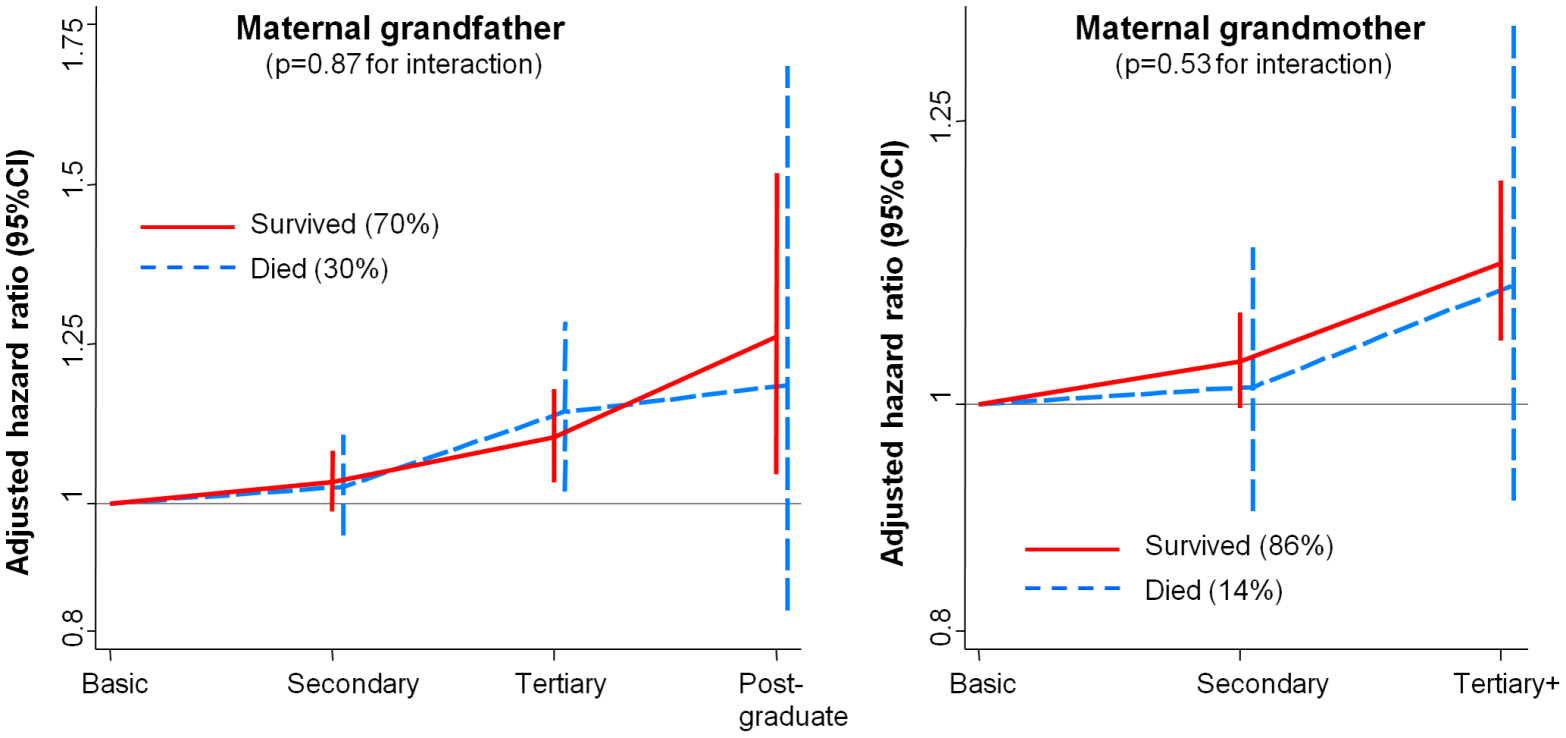
Association between maternal grandparent education and ED in granddaughters, stratifying by whether the grandparent was alive when the granddaughter turned 12 (N = 1,040,165 females). CI = confidence interval, ED = eating disorder.

Postgraduate versus basic grandparental education was associated with around a two-fold increase in crude ED rates among granddaughters. These associations were substantially attenuated after adjusting for parental education, but there remained some evidence of an independent effect of maternal grandparental education (Table 1). Again, the magnitude of these effects was similar across different ED subtypes (Table S3 in File S1). These effects of maternal grandparent education also did not vary according to whether the grandparent in question was alive when the granddaughter turned 12 (p>0.2 for interaction between education and survival for all four grandparents in both minimally-adjusted and adjusted models: see also Figure 2). As had been the case when considering parental SEP, there was no evidence of independent effects of grandparental income after adjusting for grandparental education.

In males, no grandparental SEP indicator showed significant evidence of an independent association with either anorexia nervosa or non-anorexia ED (all p>0.1), but the wide confidence intervals indicated that once again these analyses were underpowered (Table 2).

### No evidence that socio-economically advantaged groups received care for milder disorders

There was no evidence that incident inpatient cases from high SEP groups had a shorter duration of hospitalisation than those from low SEP groups (Table 3). Instead the trend was in the opposite direction, with higher SEP tending to predict a longer duration of hospitalisation (e.g. mean duration 33 vs. 28 days if any parent had vs. had not been to university). When analysing ED subtypes separately, this effect was particularly strong for anorexia nervosa (e.g. mean 36 vs. 31 days if any parent had vs. had not been to university) but the trend was also in the same direction for bulimia nervosa (30 vs. 27 days) and EDNOS (22 vs. 21 days). These findings are presented for both sexes combined; the results were very similar when excluding the small number of males. These results therefore provided no evidence that individuals from high SEP groups were accessing care for relatively more mild disorders than individuals from low SEP groups. This, in turn, gives some reason to believe that the results described in the previous section do not simply reflect individuals from high SEP families being more likely to seek healthcare or to receive treatment.

**Table 3:** Association between parent SEP and duration of first ED inpatient hospitalisation, among 5867 Swedish males and females born 1973–1998.

		N	Median duration in days	Mean duration in days	Minimally-adjusted p-value for association†
Father's	Basic	1213	10	29.9	p = 0.03
highest	Secondary	2652	11	28.5	
education	Tertiary, <3 years	775	13	33.7	
level	Tertiary, ≥3 years	1098	12	30.6	
	Post-graduate	126	12	36.4	
Mother's	Basic	805	10	26.6	p = 0.01
highest	Secondary	2800	11	28.7	
education	Tertiary, <3 years	1028	13	33.1	
level	Tertiary, ≥3 years	1198	12	32.6	
	Post-graduate	35	14	34.9	
Parents'	Unskilled manual	694	9	24.6	p = 0.09
social	Skilled manual	942	11	29.7	
class	Farmer/self-employed	746	10	26.9	
	Low non-manual	391	11	30.9	
	Medium non-manual	1521	12	32.1	
	High non-manual	1439	13	32.6	

† From linear regression predicting to log-transformed mean duration of hospitalisation, adjusting for the child's birth year, type of disorder and age at admission. Note these analyses use the duration of the first inpatient hospitalization, not excluding individuals who had previously received an outpatient diagnosis: the total number of inpatient cases is therefore higher than the 4177 cases *first* identified in the inpatient register.

## Discussion

In this study of 2 million Swedish males and females, ED incidence in daughters was independently predicted by the education of the father, the mother and the maternal grandparents. This association was observed with equal magnitude for anorexia nervosa, bulimia nervosa and EDNOS, and had become stronger over time. As for ED in males, the SEP associations appeared similar to those seen in females with respect to anorexia nervosa. By contrast, non-anorexia ED showed a different social patterning in males compared to females, being unrelated to education and being negatively associated with income. We did not find any suggestion that the offspring of high-SEP parents were hospitalised for shorter periods of time, providing tentative evidence against systematic socio-economic differences in the probability of seeking or accessing healthcare.

### Study limitations

Compared to previous large studies [5]–[7], we used a wider range of socio-economic indicators and had sufficient power, at least in females, to subdivide these more finely (e.g. distinguishing post-graduate versus tertiary-level education). Another study strength is that we compared effects across anorexia nervosa, bulimia nervosa and the rarely-studied EDNOS. On the other hand, although our study improves on most previous Swedish register studies in using outpatient as well as inpatient records for at least some of our follow-up period, it is still not community based. This is a key limitation, since community-based surveys in the USA and in Finland estimate that under 50% of individuals with ED ever access specialist ED treatment [8], [21], [22], and the proportion that receive inpatient treatment will be even lower. This key limitation means that we cannot examine how far the increased total rate of ED hospitalisation over the time period examined reflects changes in health-seeking or referral behaviours as opposed to changes in the underlying rate of pathology.

Similarly we cannot fully exclude the possibility of socio-economic biases in the probability of seeking or accessing healthcare. We believe, however, that it is implausible that such socio-economic biases explain our findings with respect to our primary aim, namely to characterise the social patterning of ED. Firstly we replicated a previous British study [6] in finding no evidence that high SEP groups were receiving care for milder disorders for any ED subtype, as judged by the (admittedly far from perfect) technique of using duration of inpatient hospitalisation as a marker for disease severity. This provides some evidence against the possibility that high SEP parents are simply more able or willing to seek care for their children. Second, one would if anything expect ED cases from high SEP groups to be under-represented not over-represented in our data, because data on outpatient discharges were much less complete for private facilities [19]. Third, healthcare in Sweden was free or low-cost at the point of delivery throughout our follow-up period, and access was comparatively equal across social groups [23]. Finally, one might expect socio-economic biases to operate similarly across the sexes and across different disorder subtypes. The fact that EDNOS in males showed a different pattern to EDNOS in females or anorexia nervosa in males therefore provides some indirect evidence against systematic biases in accessing healthcare. Similar reassurance is provided by the fact that hospitalisation rates in Sweden are higher among disadvantaged groups for most mental disorders, including depression, nonaffective psychosis and substance misuse [24].

A second important limitation of our study is that it is purely registry based, rather than linking registry data to questionnaire surveys, e.g. [25]. Although this gave us much greater power, it meant that we did not have access to data on the psychological and psychosocial characteristics that may plausibly mediate the observed associations. Although we speculate below about the reasons for these associations, further research is required to test these hypotheses.

Finally, although our inclusion of males as well as females is a study strength, in practice the number of male cases was too small to allow a meaningful examination of intergenerational effects, and several of our other analyses were also underpowered. As such, our conclusions with respect to association between family SEP and ED in males are necessarily more limited, and replication of our findings in other populations is warranted.

### Meaning of the study and directions for future research

The public health importance of understanding the aetiology of ED is underlined by our finding that rates of inpatient hospitalisation have increased over time in Sweden in both females and, more recently, in males. Although it is a limitation of this study that we cannot test whether this increase reflects changes in healthcare seeking behaviours, our findings do at a minimum indicate that ED remain an important issue for the Swedish health service. The increased hospitalisation rate observed in our data is also consistent with previous studies focussing on anxiety and depression [26], [27], and provides some tentative further evidence that some aspects of mental health have deteriorated in recent decades among adolescents and young adults in Sweden.

With regard to the role of parental SEP in the aetiology of ED in females, our findings emphatically do not mean that cases of ED are confined to the daughters of highly-educated parents. On the contrary, a larger absolute number of cases occurred among daughters of less educated parents (Table 1), because they represent a larger share of the population. Nevertheless, the higher rate of female ED in high SEP families supports three previous large studies [5]–[7] and extends these by suggesting that the same is true for anorexia nervosa in males (see also [28]).

This study also extends previous research in showing that the associations between female ED and parental SEP appear primarily to be driven by education – parental income and parental social class both showed little or no independent effect. The same appeared true with respect to anorexia nervosa in males. This specificity with respect to SEP indicator is important and intriguing, and requires further investigation. Speculatively, one possible mechanism for this effect could involve internal and external demands to succeed academically. ED symptoms and disorders, particularly those related to anorexia nervosa, are associated with both high external demands (e.g. high parental expectations) and internal demands (e.g. perfectionism, comparing oneself unfavourably with others) [25], [29]–[33]. That a drive to achieve academically may sometimes accompany and perhaps contribute to the development of ED is supported by qualitative studies [15], [34], and also by quantitative studies reporting that females with ED get higher school grades than would be expected given their IQ [35] or their parents' education [16]. One possible mechanism for the association observed in this study involves genetic confounding, with heritable parental traits such as perfectionism being associated with higher education attainment in the parents and also increased perfectionism (and therefore increased ED risk) in their offspring. We believe, however, that it is also possible that high parental education itself plays some causal role if it intensifies the magnitude of an offspring's drive for academic success. This possibility is supported by international research indicating that young people from socio-economically advantaged groups may feel both particularly motivated and under particular pressure to succeed academically [34]. One likely reason for this is the higher cultural value placed upon education in high-SEP groups [34], and this cultural value is plausibly more closely related to parents' own education than to the parents' income or occupation. This therefore offers one potential explanation for our finding that parental education seemed to be the facet of socio-economic advantage that most strongly predicted ED risk in daughters, and anorexia nervosa risk in sons.

The potential importance of the symbolic value attributed to education is further suggested by one of the most novel features of this study, namely its inclusion of grandparental characteristics. In a slightly older Swedish cohort, we have previously shown that educational outcomes are predicted by the socio-economic experiences of multiple previous generations [36]. Here we show that the same is true of a health outcome linked to high education and, moreover, that an independent effect of maternal grandparental education remains even after adjusting for parental education, income and social class. The associations with ED risk in granddaughters were seen regardless of whether the grandparent in question was alive, which we interpret as evidence against direct transmission of these effects from grandparents to their granddaughters. An explanation of genetic confounding also seems less plausible here, since these men and (particularly) women were part of a generation for whom educational attainment was less meritocratic, and relatively little predicted by individual traits such as school achievement [36]. Instead we suggest that the observed maternal grandparental effects may reflect indirect mediation via the attitudes, values or childhood experiences of the mothers. We therefore hypothesise that grandparent education may represent a further marker for the emphasis placed upon education within the family cultures of our cohort members. If true, this suggests a trade-off against the protective role which a cultural commitment to education may play with respect to more common mental health problems such as conduct and hyperactivity disorders [37]. It further highlights how SEP differences may reflect not only economic resources or material conditions, but also socio-cultural factors.

Finally, it is striking that although bulimia nervosa and EDNOS were positively associated with higher parental education in females, non-anorexia ED showed a markedly different social patterning in males. One possible explanation is suggested by the authors of one previous paper reporting on gender differences in SEP associations; they speculate that males show a different association because their ED symptoms are more likely to reflect global psychological impairments [38]. Plausibly this may apply particularly strongly to symptoms which do not meet the criteria for an ED diagnosis and therefore gain a ‘not-otherwise-specified’ diagnosis (which in practice was the case for the large majority of non-anorexia ED cases in males). If the gender differences reported in our study are replicated in other populations, then further examination of the source of these differences may shed light onto the aetiology of ED in both males and females. It may also inform the basis for preventive interventions targeting a comparatively neglected disorder (EDNOS) in this comparatively neglected group (low SEP males).

In conclusion, this research has clarified the nature of the association between parental SEP and ED in Sweden, indicating that family history of education appears to have a pervasive impact across all ED subtypes in females and perhaps upon anorexia nervosa in males. Given that these effects appear to be getting stronger over time, we believe that there is an important need for qualitative and quantitative research which can seek to elucidate the underlying mechanisms and then use these as the basis for public health interventions.

## Supporting Information

File S1 Contains Table S1, Table S2 and Table S3.
(DOC)
