# Supplementary material for: Family History of Education Predicts Eating Disorders across Multiple Generations among 2 Million Swedish Males and Females
Source: PLoS One. 2014 Aug 27;9(8):e106475. doi: 10.1371/journal.pone.0106475 (PMC4146600; doi:10.1371/journal.pone.0106475)
Supplement: File S1 — Contains Table S1, Table S2 and Table S3. (DOC) [file pone.0106475.s001.doc]

Table S1: Parental and grandparental socio-economic an age predictors of ED diagnosis among Swedish females born 1973-1998 (15 747 cases in 1 040 165 individuals): hazards ratios and 95% CI

|  |  |  | **Minimally-adjusted** | **Adjusted 1** | **Adjusted 2** | **Adjusted 3** |
| --- | --- | --- | --- | --- | --- | --- |
| Paternal | Grandfather’s | Basic | 1*** |  | 1*** | 1 |
| grand- | highest | Secondary | 1.08 (1.04, 1.13) |  | 1.05 (1.01, 1.09) | 1.01 (0.97, 1.06) |
| parents | education level | Tertiary | 1.23 (1.17, 1.29) |  | 1.11 (1.05, 1.18) | 1.02 (0.96, 1.08) |
|  |  | Post-graduate | 1.56 (1.33, 1.83) |  | 1.30 (1.10, 1.54) | 1.14 (0.97, 1.35) |
|  | Grandmother’s | Basic | 1*** |  | 1** | 1 |
|  | highest | Secondary | 1.09 (1.05, 1.13) |  | 1.05 (1.01, 1.09) | 1.04 (1.00, 1.08) |
|  | education level | Tertiary & above | 1.24 (1.17, 1.31) |  | 1.09 (1.02, 1.16) | 1.04 (0.97, 1.11) |
|  | Grandparents’ income | Change per standard deviation | 1.02 (1.01, 1.02)*** |  | 1.00 (0.99, 1.02) | 0.99 (0.97, 1.01) |
|  | Grandfather’s s age at father’s birth | Change per decade | 1.01 (0.99, 1.04) |  | 1.01 (0.97, 1.05) | 1.00 (0.97, 1.04) |
|  | Grandmother’s s age at father’s birth | Change per decade | 1.03 (1.00, 1.05) |  | 1.02 (0.97, 1.06) | 0.99 (0.95, 1.03) |
| Maternal | Grandfather’s | Basic | 1*** |  | 1*** | 1*** |
| grand- | highest | Secondary | 1.09 (1.05, 1.14) |  | 1.06 (1.02, 1.10) | 1.03 (0.99, 1.07) |
| parents | education level | Tertiary | 1.35 (1.28, 1.42) |  | 1.20 (1.14, 1.27) | 1.11 (1.05, 1.18) |
|  |  | Post-graduate | 1.73 (1.48, 2.02) |  | 1.39 (1.18, 1.63) | 1.24 (1.05, 1.45) |
|  | Grandmother’s | Basic | 1*** |  | 1*** | 1** |
|  | highest | Secondary | 1.08 (1.04, 1.12) |  | 1.04 (1.00, 1.08) | 1.03 (0.99, 1.07) |
|  | education level | Tertiary & above | 1.36 (1.29, 1.44) |  | 1.18 (1.12, 1.26) | 1.12 (1.05, 1.19) |
|  | Grandparents’ income | Change per standard deviation | 1.03 (1.03, 1.04)*** |  | 1.01 (1.00, 1.03) | 1.01 (0.99, 1.02) |
|  | Grandfather’s s age at mother’s birth | Change per decade | 1.00 (0.98, 1.03) |  | 0.97 (0.94, 1.01) | 0.97 (0.93, 1.00) |
|  | Grandmother’s s age at mother’s birth | Change per decade | 1.03 (1.01, 1.06)* |  | 1.06 (1.02, 1.10)** | 1.03 (0.99, 1.07) |
| Parents | Father’s highest | Basic | 1*** | 1*** |  | 1*** |
|  | education level | Secondary | 1.11 (1.06, 1.15) | 1.08 (1.04, 1.13) |  | 1.08 (1.03, 1.13) |
|  |  | Tertiary, < 3 years | 1.36 (1.29, 1.44) | 1.24 (1.17, 1.31) |  | 1.22 (1.15, 1.30) |
|  |  | Tertiary, ≥ 3 years | 1.56 (1.48, 1.64) | 1.32 (1.24, 1.40) |  | 1.28 (1.20, 1.36) |
|  |  | Post-graduate | 1.92 (1.71, 2.15) | 1.49 (1.32, 1.69) |  | 1.42 (1.25, 1.61) |
|  | Mother’s highest | Basic | 1*** | 1*** |  | 1*** |
|  | education level | Secondary | 1.07 (1.02, 1.12) | 1.03 (0.98, 1.09) |  | 1.03 (0.98, 1.08) |
|  |  | Tertiary, < 3 years | 1.31 (1.23, 1.39) | 1.15 (1.08, 1.22) |  | 1.12 (1.05, 1.19) |
|  |  | Tertiary, ≥ 3 years | 1.49 (1.41, 1.58) | 1.22 (1.14, 1.30) |  | 1.17 (1.09, 1.25) |
|  |  | Post-graduate | 2.27 (1.89, 2.74) | 1.63 (1.34, 1.99) |  | 1.51 (1.24, 1.83) |
|  | Parents’ income | Change per standard deviation | 1.03 (1.02, 1.03)*** | 1.01 (0.99, 1.02) |  | 1.00 (0.98, 1.02) |
|  | Parents’ social | Unskilled manual | 1*** | 1** |  | 1** |
|  | class | Skilled manual | 0.99 (0.93, 1.05) | 0.98 (0.93, 1.04) |  | 0.99 (0.93, 1.04) |
|  |  | Low non-manual | 1.03 (0.97, 1.10) | 0.99 (0.93, 1.06) |  | 0.99 (0.93, 1.05) |
|  |  | Farmer/self-employed | 1.02 (0.94, 1.10) | 0.99 (0.92, 1.07) |  | 0.98 (0.91, 1.06) |
|  |  | Medium non-manual | 1.25 (1.18, 1.32) | 1.08 (1.02, 1.14) |  | 1.07 (1.01, 1.14) |
|  |  | High non-manual | 1.44 (1.36, 1.52) | 1.09 (1.02, 1.17) |  | 1.08 (1.01, 1.15) |
|  | Father’s age at daughter’s birth | Change per decade, linear term | 1.11 (0.90, 1.32) | 0.79 (0.64, 0.96)* |  | 0.81 (0.66, 0.99)* |
|  |  | Change per decade, quadratic term | 1.00 (0.98, 1.03) | 1.05 (1.02, 1.08)** |  | 1.04 (1.01, 1.07)** |
|  | Mother’s age at daughter’s birth | Change per decade | 1.14 (1.11, 1.18)*** | 0.99 (0.94, 1.04) |  | 1.01 (0.96, 1.06) |

*p<0.05, **p<0.01, ***p<0.001 for heterogeneity. CI=confidence interval, ED=eating disorder. Basic education is up to age 16, Secondary education is up to age 18-19. Minimally-adjusted analyses adjust for child’s birth year, adjusted models 1-3 additionally include all variables in column.

Table S2: Parental predictors of ED subtypes among 574 720 Swedish females born 1985-1998: hazards ratios and 95% CI

|  |  | **Anorexia nervosa (4362 cases)** | | **Bulimia nervosa (1169 cases)** | | **EDNOS (4607 cases)** | |
| --- | --- | --- | --- | --- | --- | --- | --- |
|  |  | **Minimally-adjusted** | **Adjusted** | **Minimally-adjusted** | **Adjusted** | **Minimally-adjusted** | **Adjusted** |
| Father’s | Basic | 1*** | 1*** | 1*** | 1* | 1*** | 1** |
| highest | Secondary | 1.19 (1.08, 1.30) | 1.13 (1.03, 1.25) | 1.06 (0.90, 1.24) | 1.02 (0.87, 1.20) | 1.03 (0.95, 1.12) | 1.02 (0.94, 1.11) |
| education | Tertiary, < 3 years | 1.74 (1.56, 1.94) | 1.40 (1.25, 1.58) | 1.30 (1.06, 1.59) | 1.12 (0.90, 1.39) | 1.20 (1.08, 1.33) | 1.15 (1.03, 1.29) |
| level | Tertiary, ≥ 3 years | 2.17 (1.96, 2.41) | 1.56 (1.38, 1.77) | 1.57 (1.30, 1.90) | 1.28 (1.02, 1.61) | 1.37 (1.24, 1.52) | 1.24 (1.10, 1.39) |
|  | Post-graduate | 2.60 (2.11, 3.22) | 1.72 (1.36, 2.16) | 2.20 (1.48, 3.27) | 1.72 (1.11, 2.67) | 1.63 (1.30, 2.05) | 1.30 (1.02, 1.67) |
| Mother’s | Basic | 1*** | 1*** | 1*** | 1** | 1*** | 1*** |
| highest | Secondary | 1.18 (1.05, 1.33) | 1.09 (0.97, 1.23) | 1.29 (1.04, 1.60) | 1.28 (1.03, 1.60) | 1.05 (0.95, 1.16) | 1.04 (0.94, 1.16) |
| education | Tertiary, < 3 years | 1.73 (1.53, 1.96) | 1.30 (1.14, 1.49) | 1.77 (1.40, 2.24) | 1.58 (1.23, 2.04) | 1.20 (1.07, 1.35) | 1.14 (1.01, 1.29) |
| level | Tertiary, ≥ 3 years | 2.07 (1.83, 2.35) | 1.39 (1.21, 1.60) | 1.87 (1.48, 2.37) | 1.58 (1.21, 2.06) | 1.37 (1.22, 1.53) | 1.23 (1.08, 1.40) |
|  | Post-graduate | 2.98 (2.10, 4.22) | 1.69 (1.18, 2.44) | 2.59 (1.26, 5.33) | 1.86 (0.87, 3.96) | 2.58 (1.87, 3.57) | 2.13 (1.51, 3.00) |
| Parents’ income | Change per standard deviation | 1.03 (1.02, 1.04)*** | 1.01 (1.00, 1.03) | 1.03 (1.01, 1.04)** | 1.02 (0.98, 1.05) | 1.02 (1.01, 1.03)*** | 1.01 (0.99, 1.03) |
| Parents’ | Unskilled manual | 1*** | 1*** | 1*** | 1 | 1*** | 1 |
| social | Skilled manual | 1.12 (0.99, 1.26) | 1.10 (0.98, 1.23) | 1.04 (0.84, 1.29) | 1.02 (0.82, 1.26) | 0.94 (0.85, 1.04) | 0.94 (0.85, 1.04) |
| class | Low non-manual | 1.21 (1.07, 1.37) | 1.11 (0.98, 1.27) | 1.17 (0.93, 1.47) | 1.08 (0.85, 1.36) | 0.94 (0.84, 1.04) | 0.92 (0.82, 1.03) |
|  | Farmer/self-employed | 1.24 (1.06, 1.45) | 1.18 (1.01, 1.38) | 1.22 (0.93, 1.62) | 1.13 (0.86, 1.50) | 0.98 (0.85, 1.13) | 0.98 (0.85, 1.12) |
|  | Medium non-manual | 1.74 (1.56, 1.94) | 1.32 (1.18, 1.49) | 1.44 (1.18, 1.75) | 1.12 (0.90, 1.39) | 1.08 (0.98, 1.19) | 0.97 (0.88, 1.08) |
|  | High non-manual | 2.10 (1.88, 2.34) | 1.32 (1.15, 1.50) | 1.55 (1.26, 1.90) | 1.01 (0.78, 1.30) | 1.25 (1.13, 1.38) | 1.00 (0.88, 1.13) |

*p<0.05, **p<0.01, ***p<0.001 for heterogeneity. CI=confidence interval, EDNOS=eating disorder not otherwise specified. Basic education is up to age 16, Secondary education is up to age 18-19. Models adjust for all variables in the column, plus parents’ age, and the index cohort member’s birth year.

Table S3: Grandparental predictors of anorexia nervosa and non-anorexia eating disorder, among 952,031 Swedish females born 1975-1998: hazards ratios and 95% CI

|  |  | **Anorexia nervosa (6739 cases)** | | **Non-anorexia ED (9364 cases)** | |
| --- | --- | --- | --- | --- | --- |
|  |  | **Minimally-adjusted** | **Adjusted** | **Minimally-adjusted** | **Adjusted** |
| Highest | Basic | 1*** | 1 | 1*** | 1 |
| education | Secondary | 1.10 (1.05, 1.17) | 1.05 (0.99, 1.11) | 1.08 (1.03, 1.13) | 1.04 (0.99, 1.09) |
| of either paternal | Tertiary | 1.31 (1.22, 1.40) | 1.03 (0.96, 1.11) | 1.19 (1.12, 1.27) | 1.07 (1.00, 1.14) |
| grandparent | Post-graduate | 1.62 (1.27, 2.05) | 1.05 (0.83, 1.34) | 1.49 (1.21, 1.84) | 1.22 (0.98, 1.51) |
| Paternal grand-parents’ income | Change per standard deviation | 1.02 (1.01, 1.03***) | 1.00 (0.98, 1.02) | 1.01 (1.00, 1.02)** | 0.99 (0.96, 1.01) |
| Highest | Basic | 1*** | 1** | 1*** | 1*** |
| education | Secondary | 1.04 (0.98, 1.09) | 0.99 (0.94, 1.05) | 1.13 (1.08, 1.18) | 1.10 (1.05, 1.15) |
| of either maternal | Tertiary | 1.36 (1.27, 1.46) | 1.09 (1.02, 1.17) | 1.35 (1.28, 1.44) | 1.23 (1.16, 1.31) |
| grandparent | Post-graduate | 1.95 (1.57, 2.43) | 1.29 (1.03, 1.62) | 1.63 (1.32, 2.01) | 1.36 (1.10, 1.69) |
| Maternal grand-parents’ income | Change per standard deviation | 1.04 (1.03, 1.05)*** | 1.02 (1.00, 1.03) | 1.03 (1.02, 1.04)*** | 1.00 (0.98, 1.02) |

*p<0.05, **p<0.01, ***p<0.001 for heterogeneity. CI=confidence interval. Models adjust for all variables in the column, plus the grandparents’ income and age, parents’ education, income, social class and age, and the index cohort member’s birth year. Note that these analyses divide ED into anorexia nervosa and non-anorexia ED because this division is possible in ICD-9 as well as ICD-10, and this therefore increases the sample size for these less-well-powered inter-generational analyses. For the same reason, these analyses combine grandmothers and grandfathers, since these showed similar associations.
